# Discerning the role of polymyxin B nonapeptide in restoring the antibacterial activity of azithromycin against antibiotic-resistant *Escherichia coli*

**DOI:** 10.3389/fmicb.2022.998671

**Published:** 2022-09-21

**Authors:** Farah Al-Marzooq, Akela Ghazawi, Saeed Tariq, Lana Daoud, Timothy Collyns

**Affiliations:** ^1^Department of Medical Microbiology and Immunology, College of Medicine and Health Sciences, United Arab Emirates University, Al Ain, United Arab Emirates; ^2^Department of Anatomy, College of Medicine and Health Science, United Arab Emirates University, Al Ain, United Arab Emirates; ^3^Tawam Hospital, Al Ain, United Arab Emirates

**Keywords:** polymyxin B nonapeptide, azithromycin, *Escherichia coli*, antimicrobial resistance, synergy

## Abstract

Antimicrobial resistance is a global public health threat. Antibiotic development pipeline has few new drugs; therefore, using antibiotic adjuvants has been envisioned as a successful method to preserve existing medications to fight multidrug-resistant (MDR) pathogens. In this study, we investigated the synergistic effect of a polymyxin derivative known as polymyxin B nonapeptide (PMBN) with azithromycin (AZT). A total of 54 *Escherichia coli* strains were first characterized for macrolide resistance genes, and susceptibility to different antibiotics, including AZT. A subset of 24 strains was then selected for synergy testing by the checkerboard assay. PMBN was able to re-sensitize the bacteria to AZT, even in strains with high minimum inhibitory concentrations (MIC: 32 to ≥128 μg/ml) for AZT, and in strains resistant to the last resort drugs such as colistin and meropenem. The fractional inhibitory concentration index was lower than 0.5, demonstrating that PMBN and AZT combinations had a synergistic effect. The combinations worked efficiently in strains carrying *mphA* gene encoding macrolide phosphotransferase which can cause macrolide inactivation. However, the combinations were inactive in strains having an additional *ermB* gene encoding macrolide methylase which causes ribosomal drug target alteration. Killing kinetics study showed a significant reduction of bacterial growth after 6 h of treatment with complete killing achieved after 24 h. Transmission electron microscopy showed morphological alterations in the bacteria treated with PMBN alone or in combination with AZT, with evidence of damage to the outer membrane. These results suggested that PMBN acted by increasing the permeability of bacterial outer membrane to AZT, which was also evident using a fluorometric assay. Using multiple antimicrobial agents could therefore be a promising strategy in the eradication of MDR bacteria. PMBN is a good candidate for use with other antibiotics to potentiate their activity, but further studies are required *in vivo*. This will significantly contribute to resolving antimicrobial resistance crisis.

## Introduction

Antimicrobial resistance (AMR) is a global public health crisis (Murray et al., 2022). The World Health Organization (WHO) considered multidrug-resistant (MDR) Gram-negative bacteria (GNB) as a serious threat forebodes returning to a pre-antibiotic era. In the era of coronavirus disease 2019 (COVID-19), managing infections is considered a top priority, especially that this pandemic has been accompanied by increased consumption of antibiotics (Lucien et al., 2021). Limited therapeutic agents are currently effective in treating infections caused by MDR bacteria; therefore, new therapeutic strategies are required (Hamad et al., 2019). It is feasible to further investigate synergistic treatments in solving the AMR crisis, as combinations of multiple antimicrobial agents have proven to be effective; for example, multidrug therapy for managing sepsis caused by carbapenem-resistant *Enterobacteriaceae* in critically ill patients (Ahmed et al., 2014). The combination of different drugs offers many advantages over the use of a single agent. These advantages include dosage reduction, lower risk for AMR, synergistic effects and the ability to attack multiple bacterial targets simultaneously, leading to an enhanced antibacterial effect (León-Buitimea et al., 2020).

*Enterobacterales* (including *Escherichia coli*) are among the most common GNB that are resistant to many antibiotics (Stapleton et al., 2020). *E. coli* is a member of the normal intestinal microbiota in humans (Kaper et al., 2004). It is responsible for a variety of diseases in both community and hospital settings, associated with high rates of morbidity and mortality (Wu et al., 2021). *E. coli* is the single most important causative agent of urinary tract infections (UTI), accounting for more than 80% of the UTI episodes (Foxman, 2010). Besides, it can cause a variety of diseases such as bloodstream infections, gastrointestinal infections, sepsis and meningitis (Kaper et al., 2004). Azithromycin (AZT) is a macrolide antibiotic which inhibits bacterial protein synthesis (Parnham et al., 2014). Macrolides, and AZT in particular, have a number of reported non-bactericidal properties that could further complement their antibacterial efficacy. AZT is touted to have anti-inflammatory and immunoregulatory properties through modulation of innate and adaptive immune responses (Zimmermann et al., 2018); thus, it has been used to treat chronic inflammatory disorders of the respiratory system (Zarogoulidis et al., 2012). Furthermore, it has antiviral properties with proven *in vitro* activity against several viruses like rhinovirus (Schögler et al., 2015) and influenza virus (Tran et al., 2019). AZT antiviral activity was attributed to its ability to reduce the viral entry into the host cells (Tran et al., 2019; Du et al., 2021), and through indirect mechanisms relying on its anti-inflammatory activity (Mohanta et al., 2020; Poddighe and Aljofan, 2020).

In general, macrolides have low levels of activity against *Enterobacterales* which have been linked to the poor membrane penetration (Gomes et al., 2017). Several studies suggested that AZT has synergistic properties in combination with other antimicrobial agents on GNB (Fernández-Cuenca et al., 2003; Wu et al., 2017), including *E. coli* (Li et al., 2018). In the latter study, AZT was used in combination with colistin, which acts by destroying the outer membrane of GNB, thereby increasing the amount of AZT in the cytoplasm. A major limitation of the previous studies is the lack of correlation with the genomic properties of the bacteria, as genes responsible for resistance to macrolide were not tested. Some studies reported that colistin-based combination therapy may increase toxicity in the kidneys and liver (Bassetti et al., 2008); thus, more safe alternatives are needed. One of the derivatives of polymyxin is polymyxin B nonapeptide (PMBN), a cationic cyclic peptide derived by enzymatic processing from the naturally occurring peptide polymyxin B. Like colistin, it can increase the permeability of the outer membrane (OM) of GNB toward hydrophobic antibiotics probably by binding to the bacterial lipopolysaccharide (LPS; Tsubery et al., 2000). PMBN is less toxic, lacks bactericidal activity, but is thought to retain its ability to render GNB susceptible to several antibiotics by permeabilizing their outer membranes (Vaara, 2013). Previous studies have demonstrated success in combining polymyxin derivatives with other antibiotics, but mechanistic studies or correlation with genotype of the tested strains were not reported (Vaara, 2019). This study aimed to test the possibility of enhancing the antibacterial activities of AZT in combination with PMBN by investigating their effect on a group of *E. coli* strains characterized for macrolide resistance genes. The study also aimed to determine the killing kinetics and to examine the effect of the combinations on the outer bacterial membrane with detection of the ultrastructural changes in the treated bacterial cells by the use of transmission electron microscopy.

## Materials and methods

### Bacterial strains

In this study, a total of 54 *E. coli* strains were investigated, including three bacterial strains obtained from the American Type Culture Collection (ATCC) purchased from Microbiologics, United States, namely, ATCC BAA-2469, CDC AR-0346, and ATCC 25922. In addition, 51 clinical strains isolated from patients attending to Tawam Hospital, Al-Ain, UAE were tested (Supplementary Table S1). Bacterial identification was performed using VITEK 2 system. All isolates were preserved in brain heart infusion broth (MAST, United Kingdom) containing 20% glycerol and stored in −80°C freezer. The strains were checked for purity before any experiment.

### Antibiotic susceptibility testing

Antibiotic susceptibility testing was performed according to the Clinical Laboratory Standards Institute (CLSI) guidelines using *E. coli* ATCC25922 as a quality control (Clinical and Laboratory Standards Institute, 2020). Disk diffusion test was used for the assessment of susceptibility to amoxicillin/clavulanate, cefpodoxime, ceftazidime, cefotaxime, cefepime, cefoxitin, aztreonam, gentamicin, piperacillin/tazobactam, ciprofloxacin, imipenem, ertapenem, meropenem, and co-trimoxazole. Antibiotic disks were obtained from MAST, UK and were applied on Mueller–Hinton agar plates (Oxoid, United Kingdom). Broth microdilution test was used to estimate the minimum inhibitory concentration (MIC) for selected antibiotics, including ceftazidime, ceftazidime-avibactam, cefotaxime, aztreonam, meropenem, ciprofloxacin, gentamicin, amikacin, colistin, azithromycin, and polymyxin B nonapeptide (PMBN). Antibiotic powders were purchased from Sigma-Aldrich, United States. Mueller–Hinton broth obtained from Oxoid, United Kingdom, was used for the assessment of MICs in accordance with CLSI guidelines (Clinical and Laboratory Standards Institute, 2020).

Based on antibiotic susceptibility profiles, the strains were classified as multidrug resistant (MDR) if they were resistant to at least one antibiotic in three or more antimicrobial categories (Magiorakos et al., 2012), and extensively drug resistant (XDR) if they were resistant to one antibiotic in ≥6 antimicrobial categories (Saderi and Owlia, 2015).

### Genotyping—detection of resistance genes

Detection of macrolide resistance genes including genes encoding phosphotransferases (*mphA* and *mphB*), methylases (*ermA*, *ermB*, and *ermC*), esterase (*ereA*), and efflux pumps (*msrA, msrD*, *mefA*, and *mefB*) was done by PCR, as described previously (Gomes et al., 2019). PCR was also used to detect major carbapenemase genes (*bla*_NDM_, *bla*_OXA-48-like_, *bla*_KPC_, *bla*_VIM_, *bla*_IMP_), and extended spectrum beta-lactamase (ESBL) genes (*bla*_TEM_, *bla*_CTX-M_, *bla*_SHV_, *bla*_OXA-1_) using primers and conditions previously described (Al-Marzooq et al., 2015).

### Checkerboard synergy assay

To evaluate the synergism between PMBN and AZT, checkerboard assay was implemented. Synergy testing was carried out in 96-well microtiter plates on an initial inoculum of 5 × 10^5^ CFU/ml using a 7 × 5 well configuration with a final volume of 100 μl (Antonello et al., 2021). AZT was serially diluted starting from a concentration which is two times higher than the MIC (if the bacteria was susceptible), and at 128 μg/ml if the strain was resistant. To date, breakpoints for PMBN as an antibacterial agent are not available, so 32 μg/ml was tested as the maximum dose in the checkerboard assay. MICs of the drugs alone and in combination were determined as the lowest drug concentration inhibiting bacterial growth following overnight incubation at 37°C. As a measure of synergy, fractional inhibitory concentration index (FICI) was calculated. FICI represents the sum of the FICs of each drug tested. The FIC for each drug is determined by dividing the MIC of each drug when used in combination by the MIC of each drug when used alone, as follows:

FICI = (MIC_AA + B_/MIC_A_) + (MIC_BA + B_/MIC_B_).

MIC_A_ and MIC_B_ denote the MIC of each drug alone, and MIC_AA + B_ and MIC_BA + B_ represent the concentrations of drug A and B in the combination.

Though PMBN was not active as an antibiotic, its MIC was considered as 128 μg/ml (highest concentration tested) for FICI calculation for most of the strains except those with determined MIC. This was done by other investigators if the tested compounds were not active when used alone (Yarlagadda et al., 2020). The combination was considered synergistic when the FICI was ≤0.5, no interaction occurred when the FICI was >0.5 and ≤ 4, and an antagonistic effect was considered when the FICI was >4 (Rattanapanadda et al., 2019).

### Time-kill study

Based on the checkerboard assays, the combinations showing synergy were tested for their killing kinetics in five selected strains (White et al., 1996). Time-kill curves were used to monitor bacterial growth and death over a wide range of antimicrobial concentrations. Bacteria were grown overnight in Trypton Soy agar (TSA) at 37°C. Bacterial cultures in saline were diluted in Mueller–Hinton broth (Oxoid, United Kingdom) to an inoculum of 5 × 10^5^ CFU/ml. Drugs were applied as described for the checkerboard assay. Time-kill curves were obtained by removing aliquots from wells with the AZT/PMBN combinations and those with a single agent applied to the bacterial strains grown in a 96-well plate. The plates were incubated at 37°C, and aliquots were collected at different time points (0, 2, 4, 6, and 24 h). At each time point, an inoculum of 5 μl was taken from each well and 10-fold serially diluted in 0.85% NaCl. Immediately after the dilution, 5 μl drop was pipetted on TSA plate and incubated at 37°C overnight for the enumeration of colony forming units (CFU) in each combination (Wu et al., 2019). The detection limit of the assay was 200 CFU/ml, if a minimum growth of one colony was obtained on a TSA platted with the least sample dilution (5 μl aliquot from 100 μl culture). A CFU of 0 was reported if no growth was observed from the least dilution tested. The procedure was performed in duplicate (two independent experiments) for each strain using round bottom microwell plates (Sarstedt, Germany) for proper mixing during dilution. A graph was plotted using log_10_ bacterial concentration at each time point for each concentration tested. Log_10_ CFU/ml was plotted on the Y axis against time on X axis (Hamad et al., 2022).

### Outer membrane permeability

The outer membrane permeation activity of the PMPN and combinations was assessed by the 1-N-phenylnaphthylamine (NPN) assay (Sigma, United States), as described previously with slight modifications (Zhou et al., 2019). Briefly, mid-logarithmic phase bacterial cells adjusted to a density of 10^9^ CFU/ml (equivalent to 3.0 MacFarland) were added to black 96 well microplates containing 10 μM NPN and colistin, PMBN or AZT (serial dilutions), or a combination of PMBN and AZM (at FICI). The plates were incubated for 1 h at 37° C protected from light. Fluorescence intensity was measured after 1 h using infinite M200 PRO fluorescence microplate reader (Tecan, United States) at 350 nm excitation and 420 nm emission wavelengths. NPN uptake (%) was calculated for each strain as described before (MacNair et al., 2018), as follows:

NPN uptake (%) = (*F*_obs_ − *F*_0_) / (*F*_100_ − *F*_0_) × 100%.

Where *F*_obs_ is the observed fluorescence at a given concentration of the drug, *F*_0_ is the initial fluorescence of NPN with *E. coli* cells in the absence of any treatment, and *F*_100_ is the fluorescence of NPN with *E. coli* cells upon addition of 128 μg/ml of colistin, as full NPN uptake (100%) was reported to be achieved at high concentrations of colistin (MacNair et al., 2018; Zhou et al., 2019).

### Transmission electron microscopy

Transmission electron microscopy (TEM) was used to look for changes in cell morphology of a selected bacterial strain (EC26) after treatment with the test agents. The procedure followed as described before with some modifications (Nguyen et al., 2021). A single colony from an overnight culture was suspended in 10 ml LB broth (Invitrogen, United States), and incubated with shaking at 37°C overnight. The overnight culture was diluted 1:30 in 40 ml LB broth and kept under continuous agitation for 2 h, adjusted to OD_600_ of 0.5 (approximately 4 × 10^8^ CFU/ml). The bacteria were treated with either AZT (8 μg/ml); PMBN (16 μg/ml) or a combination of PMBN and AZT (16 and 8 μg/ml, respectively). Untreated culture was used as a growth control (negative control) and culture treated with colistin (2 μg/ml) was used as a positive control. Both treated and untreated cultures were incubated for 1 h with shaking at 37°C. The cells were harvested by centrifugation at 4°C to avoid cell damage, washed and resuspended in 0.1 M phosphate-buffered saline (PBS; pH 7.2). Cells were fixed overnight with a fixative containing 4% formaldehyde, 1.25% glutaraldehyde, 4% sucrose, 0.01 M CaCl_2_, 0.075% ruthenium red in PBS. The fixed cells were washed three times in PBS buffer, post fixed in 1% osmium tetroxide in PBS containing 0.075% ruthenium red for 1 h, and washed with water afterwards. Cells were dehydrated with ascending ethanol grades (30, 50, 70, 80, 90, 95, and 100%) each for 15 min, and finally treated with propylene oxide. The cells were infiltrated for 1 h each in propylene oxide: Agar 100 epoxy resin in ratio of 1:1, 1:2, and 1:3 and polymerized at 65° C for 24 h. Blocks were trimmed and then semithin and ultrathin sections were cut with EM UC7 Ultracuts ultramicrotome (Leica, Vienna, Austria). Semithin sections (1.5 μm thickness) were collected and stained with 1% aqueous toluidine blue on glass slides with a hot plate at 70°C. Ultrathin sections (95 nm) with gold color were collected on 200 mesh copper grids, air dried and then were contrasted with uranyl acetate followed by lead citrate. Then, grids were examined with transmission electron microscope (Tecnai Biotwin Spirit G2, The Netherlands) and images were taken at 80 Kv at different magnifications.

### Statistical analyses

All statistics were performed using IBM SPSS (version 26, SPSS Inc., Chicago, IL, United States). Graphs were generated using GraphPad Prism® Version 9.0 (GraphPad Software, Inc., La Jolla, CA, United States). Kruskal–Walli’s rank sum test and Mann–Whitney U-tests were performed to compare the groups as appropriate. Statistical significance was determined at *p* < 0.05.

## Results

Except the quality control strain (ATCC 25922), all the *E. coli* strains tested in this study were highly resistant to multiple antibiotics (MDR), including strains that were also resistant to the last resort drugs including carbapenems (six strains with *bla*_NDM_ gene and two strains with *bla*_OXA-48 like_ gene), and colistin (one strain with *mcr-1* gene; CDC AR-0346). Results of antibiotic susceptibility testing and resistance genes detected in the strains are shown in the Supplementary Table S1.

For macrolide resistance genes, 62.96% (*n* = 34) of the strains harbored the *mphA* gene (MIC: 32 to ≥128 μg/ml), from them 14.8% (*n* = 8) harbored the *ermB* gene in addition to the *mphA* gene and exhibited very high MICs for AZT (>128 μg/ml). On the other hand, all the 20 strains (37.04%) with low MICs for AZT (2–8 μg/ml) lacked these genes.

After defining the properties of the strains, a subset was selected for synergy testing including 24 strains with different MICs for AZT and different profiles of resistance genes. As shown in Table 1, combinations of PMBN and AZT acted in synergy (FICI < 0.5) in 16 strains including 12 strains with *mphA* gene alone. On the other hand, no synergy between the AZT with PMBN was noted in the presence of *ermB* with *mphA* in all the eight tested strains.

**Table 1:** List of strains tested using checkerboard assay for combinations of PMBN and AZT.

Strain ID	Genotype	Important characteristics—resistance profile^■^	AZT MIC (μg/ml)	PMBN MIC (μg/ml)	Effective combinations in FICI (AZT/PMBN μg/ml)	FICI
ATCC 25922	None*	Susceptible	4	>128	0.25/8	0.13
EC24	None	MDR	4	>128	0.5/8	0.19
EC520	None	CRE-MDR	8	>128	0.5/8	0.13
EC543	None	CRE-MDR	8	>128	1/4	0.16
BAA-2469	*mphA*	CRE-MDR	32	>128	8/2	0.27
EC54	*mphA*	MDR-CRE	32	>128	8/0.5	0.25
EC55	*mphA*	MDR	32	>128	8/0.5	0.25
EC14	*mphA*	MDR	32	>128	8/0.5	0.25
EC125	*mphA*	MDR	32	>128	8/0.5	0.25
EC133	*mphA*	MDR	32	>128	8/0.5	0.25
CDC AR-0346	*mphA*	XDR- colistin resistant	32	>128	8/4	0.28
EC13	*mphA*	MDR	64	>128	8/1	0.13
EC122	*mphA*	MDR	64	>128	8/4	0.14
EC477	*mphA*	MDR	64	64	8/2	0.16
EC26	*mphA*	MDR	128	>128	16/8	0.19
EC499	*mphA*	CRE-MDR	>128	64	16/8	0.19
EC162	*mphA + ermB*	MDR	>128	>128	NA #	NA
EC405	*mphA + ermB*	MDR	>128	>128	NA	NA
EC413	*mphA + ermB*	MDR	>128	>128	NA	NA
EC415	*mphA + ermB*	MDR	>128	>128	NA	NA
EC456	*mphA + ermB*	MDR	>128	>128	NA	NA
EC469	*mphA + ermB*	MDR	>128	>128	NA	NA
EC500	*mphA + ermB*	CRE-MDR	>128	128	NA	NA
EC522	*mphA + ermB*	CRE-MDR	>128	>128	NA	NA

Results of synergy are shown with genotypes (macrolide resistance genes detected in the strains) and antibiotic resistance profile.

^■^MDR, multidrug resistant; CRE, carbapenem resistant; XDR, extensively drug resistant.

* Negative for macrolide resistance genes, and susceptible to AZT.

# NA: not applicable (no synergy)

**Figure 1:**
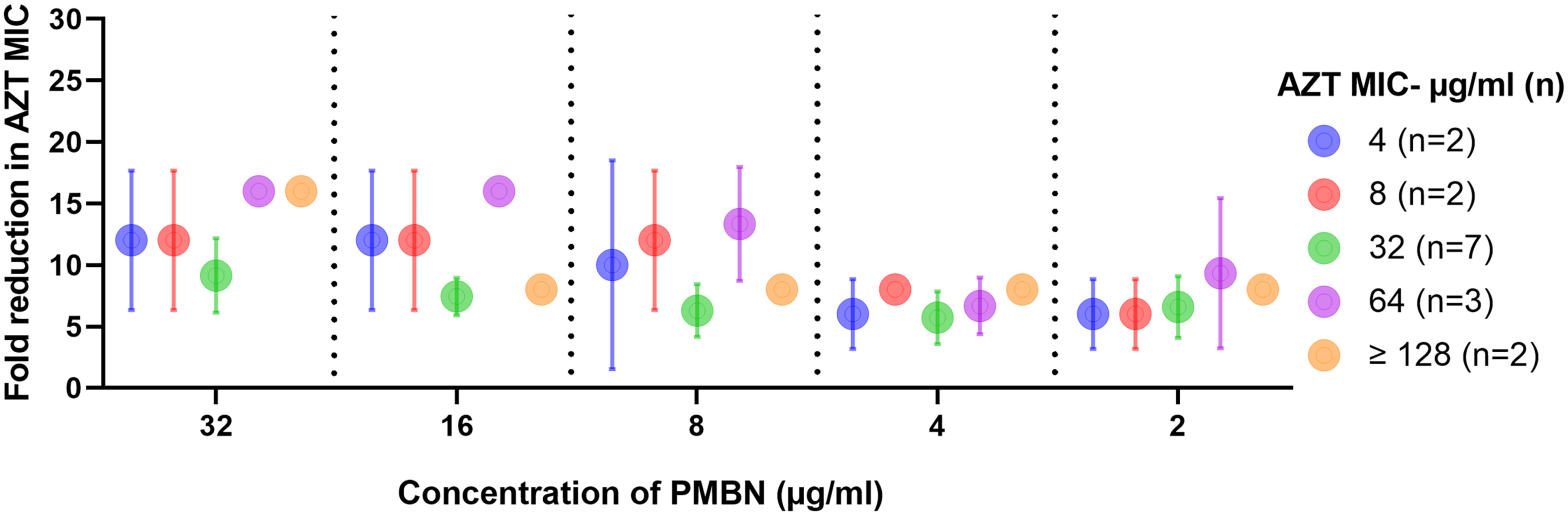
Fold reduction in AZT MIC when used in combination with different concentrations of PMBN. Data shown represent mean ± SD of fold reduction in MICs for tested strains treated with the synergistic combinations. Baseline AZT MICs (when used alone) for the strains are shown in the figure legend. Only strains (*n* = 16) against which the combinations were synergistic are shown.

The results of the checkerboard synergy assays are presented in Supplementary Table S2, which shows the results obtained from the 16 strains on which AZT and PMBN combinations were synergistic, and fold reduction in AZT MIC when used in combination with different PMBN concentrations. Supplementary Table S3 includes the mean MICs of AZT at different PMBN concentrations tested in the combinations in strains grouped based on their baseline MIC of AZT. These results are summarized in Figure 1 which shows the fold reduction in AZT MIC, when used in combination with different concentrations of PMBN on the 16 strains against which the combinations were synergistic.

In all the combinations, there was a significant decrease in the MICs of AZT, with at least 6 folds reduction (*p* < 0.05). When the mean MICs of AZT in the combinations were compared, they were not significantly different when different concentrations of PMBN were applied (*p* > 0.05), although the highest fold-reduction in MIC (9.1–16 folds) was found when the highest concentration of PMBN (32 μg/ml) was used. However, when strains with different MICs for AZT (4- ≥ 128 μg/ml) were compared, a significant difference in the MICs in the combinations was found (*p* < 0.05), as strains with lower MICs for AZT (4–8 μg/ml) demonstrated better response to treatment with AZT and PMBN (mean MIC: 0.4–1.5 μg/ml; mean folds reduction in MIC: 6–12) compared to strains having high AZT MICs (32 to ≥128 μg/ml) exhibiting mean MIC of 3–16 μg/ml (mean folds reduction in MIC: 5.7–16) when treated with both AZT and PMBN (data shown in Supplementary Table S3, and folds reduction in MICs are shown in Figure 1).

**Figure 2:**
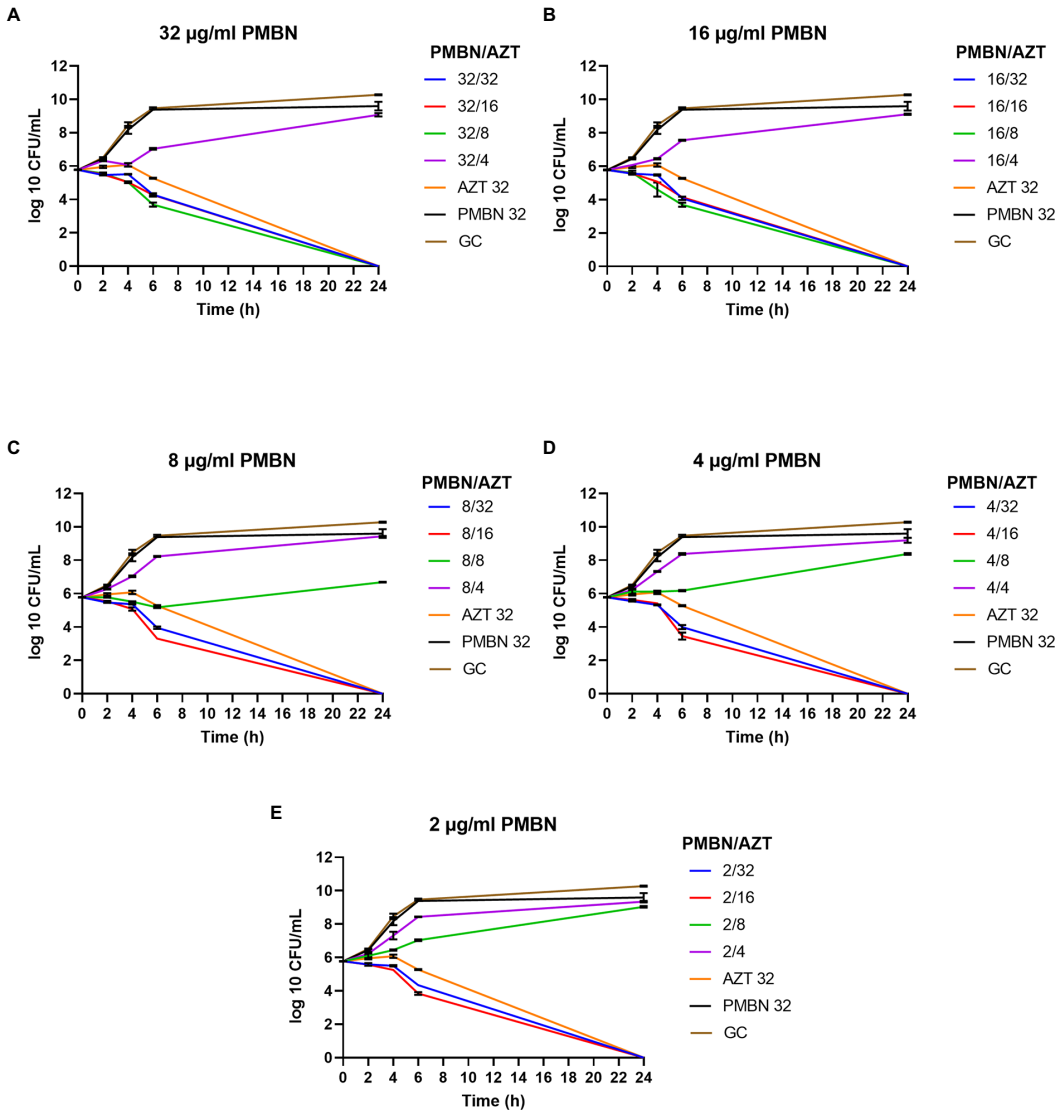
Time-kill graphs for a selected strain (*E. coli* CDC AR-0346) tested with different synergistic combinations of PMBN (2–32 μg/ml) and AZT (4–32 μg/ml). A single agent (AZT or PMBN) at a concentration of 32 μg/ml were used as controls in addition to an untreated growth control (GC). AZT ≥ 8 μg/ml killed the bacteria in all the synergistic combinations with PMBN. Mean of duplicates from two independent experiments ± SD are shown.

Time kill assays were performed for five selected *E. coli* strains, in order to determine the time required to achieve bacterial killing by the synergetic combinations. Different combinations of AZT with PMBN were tested. Multiple combinations significantly reduced the growth during the first 6 h of treatment with complete killing achieved after 24 h of treatment in all the tested strains. Notably, all the bacterial strains were highly resistant to PMBN when used alone, and to AZT when used in sub-inhibitory concentrations. Figure 2 is a representative time-kill graph for a selected strain (CDC AR-0346), while time-kill graphs of the other four strains are shown in the Supplementary Figures 1–4. The sole effect of AZT on bacterial growth was tested in one strain (EC26), and the results showed that AZT was not able to kill the bacteria at concentrations below the MIC (Supplementary Figure 4F); thus, the experiment was not attempted on more strains.

As shown in Figure 2, combinations of PMBN and AZT caused significant reduction in the bacterial growth after 6 h of treatment and complete killing after 24 h. At high concentration of PMBN (32 and 16 μg/ml; Figures 2A,B), combination with AZT (> 4 μg/ml) caused bacterial killing, while lower concentrations of PMBN (8, 4 and 2 μg/ml; Figures 2C,E), in combination with higher concentration of AZT (> 8 μg/ml) were required to achieve the same effect. When applied alone, PMBN at the highest tested concentration (32 μg/ml) had no effect on the bacteria with a growth pattern similar to the untreated growth control (Figures 2A–E). The same pattern of killing was observed in the other strains (Supplementary Figures 1–4) as PMBN concentration of 16–32 μg/ml caused killing at AZT concentration 2 folds higher than the bactericidal concentration used with 2–8 μg/ml of PMBN.

**Figure 3:**
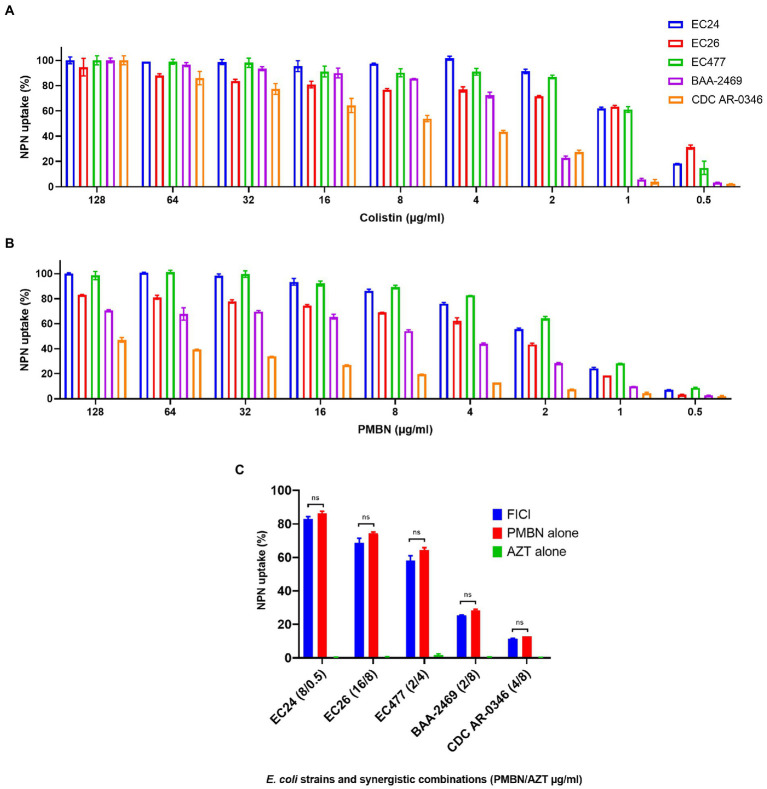
NPN uptake (%) of five selected *E. coli* strains. Data shown represent NPN uptake by bacteria treated with serial dilution (128–0.5 μg/ml) of colistin used as a positive control **(A)** and PMBN **(B)**, in addition to bacteria treated with synergetic combination of PMBN and AZT at FICI compared to treatment with a single agent **(C)**. NS is non-significant.

As shown in Figure 3 which summarizes the NPN assay results, PMBN was able to increase the permeability of the outer membrane in a concentration-dependent manner (Figure 3B), as also shown for colistin (Figure 3A) based on NPN uptake (%) after treatment with various concentrations of the drugs. AZT alone did not have any effect on the outer membrane as mean ± SD of % NPN uptake was 0.53 ± 0.3 (as shown in Figure 3C, for selected strains). Based on NPN uptake (%) results, combination of PMBN and AZT increased outer membrane permeability which was slightly less than using PMBN alone; however, the difference was not significant (Figure 3C).

It is important to note, that one strain (CDC AR-0346) which was resistant to colistin, demonstrated the least response to PMBN, compared to the other strains. Nevertheless, synergistic combinations of PMBN and AZT had bactericidal effects as shown in the time-kill study (Figure 2). As for strain CDC AR-0346, FICI (PMBN/AZT = 4/8 μg/ml) was able to lower bacterial growth significantly without killing based on the results of the time-kill study shown in Figure 2D, as the damage to the outer membrane was low (less NPN uptake) as seen in Figure 3C.

**Figure 4:**
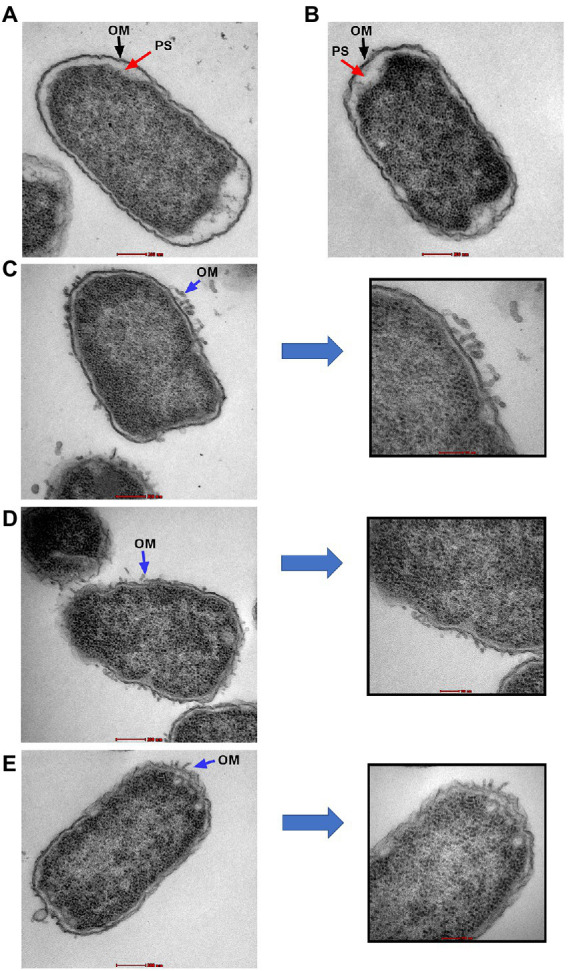
Transmission electron microscopy images of *E. coli* strain (EC26). Untreated cells **(A)** and cells treated with AZT—8 μg/ml **(B)**, are shown with intact outer membrane (OM) and clear periplasmic space (PS). Damage to the OM was seen in bacterial cells treated with colistin—2 μg/ml **(C)**, PMBN alone—16 μg/ml **(D)** and PMBN—16 μg/ml plus AZT—8 μg/ml **(E)**. Parts **(C–E)** included magnified sections to demonstrate OM damage caused by the treatment.

**Figure 5:**
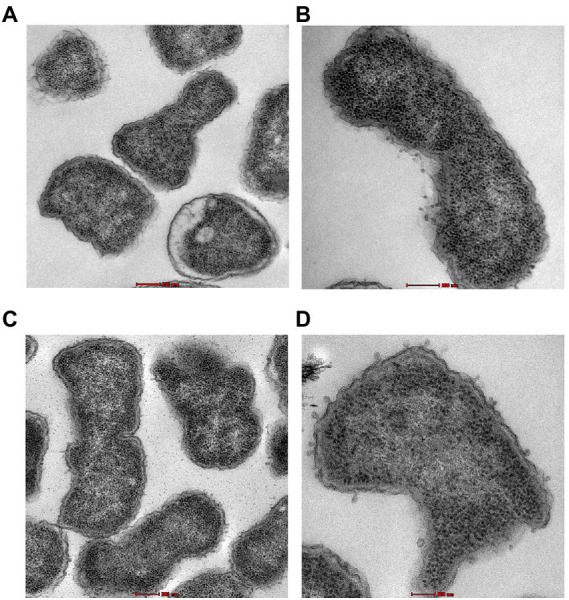
Morphological changes in the bacterial cells (*E. coli* strain EC26) treated with PMBN alone **(A, B)** and PMBN with AZT **(C, D)** observed using transmission electron microscopy.

Transmission electron microscopy was used to examine the ultrastructural changes in a selected strain (EC26) treated with the synergistic combination compared to a single agent. As shown in Figure 4, untreated bacteria (A) and bacterial cells treated with AZT (B) had intact outer membrane (OM) and a clear periplasmic space (PS). One the other hand, bacteria treated with the control antibiotic colistin (C) had disrupted OM with threads detached due to the damage. PMBN also compromised the integrity of the OM when used alone (D) as well as in combination with AZT (E). This was accompanied by loss or reduction of the periplasmic space underneath the OM. Interestingly, cells treated with PMBN alone or in the combination demonstrated alterations in the cellular morphology with loss of the characteristic rod shapes and smooth well-defined OM as seen in many cells, shown in Figure 5. Protrusions or blebs were also detected on the surface of treated *E. coli* as shown in Figures 4D–E, 5A–D demonstrating the damage to OM in a strain (EC26) treated with PMBN alone and in combination with AZT.

## Discussion

The emergence of AMR coupled with the slow development of new antimicrobial drugs represent a growing worldwide challenge for infection management which is now a top priority in the era of COVID-19 pandemic (Bendala Estrada et al., 2021). One strategy to tackle the AMR problem was thought to be by teaching old antibiotics new tricks to continue their use and preserve effectiveness against MDR bacteria (Deshayes et al., 2016). Thus, one of the approaches to fight MDR bacteria is the discovery of potent synergistic combinations, which could be implemented without the need to modify existing drugs (León-Buitimea et al., 2020). In this study, we have proven the effectiveness of synergistic combinations composed of AZT and PMBN. These combinations were found effective on MDR strains of *E. coli*, even those resistant to the last resort drugs including carbapenems and colistin. The spread of resistance to the latter drugs increased the need for alternatives to these medications to fight highly resistant bacteria (Janssen et al., 2020; Humphrey et al., 2021).

Synergy is commonly found in combinations utilizing drugs that target the bacterial membrane and antibiotics that target the biosynthesis of nucleic acids and proteins (Zharkova et al., 2019). Thus, a membrane targeting agent (PMBN) and a protein synthesis inhibitor (AZT) were selected for use in this study. AZT was selected for use in the synergetic combinations, as AZT is recognized as one of the effective weapons to fight microbial infections, augmented by its anti-inflammatory and immunomodulatory effects (Zimmermann et al., 2018). A recent multicenter study in Spain, analyzed the use of antibiotics in patients hospitalized due to SARS-CoV-2 infection. Higher mortality rates were reported with use of all antibiotics except macrolides, particularly AZT, which caused a significantly higher survival rate among COVID-19 patients (Bendala Estrada et al., 2021). It is important to note that AZT belongs to the parent class of macrolides that are characteristically bacteriostatic, with some evidence suggesting that bactericidal killing can be achieved through concentration-dependent effects (Dorfman et al., 2008). We have noticed the same in our study, as high concentrations of AZT were required to kill the bacteria when used alone, as seen in the time-kill assays. Importantly, bactericidal effects of the combinations were obtained when concentrations lower than the MIC of AZT were used, indicating the success of the combinations to lower the dose of the drug.

Previous studies have demonstrated that AZM entry and activity against MDR-GNB was synergistically enhanced when the bacterial outer membrane is perturbed by other molecules such as cationic human antimicrobial peptide (LL-37) or by the last-line antibiotic colistin (Lin et al., 2015). However, it is important to mention that the latter two drugs have antibacterial activities when used alone as reported by several investigators (Humphrey et al., 2021; Ridyard and Overhage, 2021). Added to that, colistin is well known for its side effects, such as nephrotoxicity and neurotoxicity (Poirel et al., 2017), which might be a factor for considering other alternatives with less side effects. The combination of LL-37 and AZT was also shown to increase the permeability of MDR-GNB, by initiating a positive feedback loop that increases the active intracellular levels of AZT (Lin et al., 2015).

Although polymyxin derivatives have been used to sensitize GNB to other antibiotics (Vaara, 2019), PMBN was not widely used in combination with other drugs, probably due to its weak antibacterial effects when used alone (Ferrer-Espada et al., 2019). We agree with the previous reports, as all the strains tested in this study expressed low susceptibility to PMBN monotherapy, but was effective when used in combination with AZT. It was reported that colistin can impact the surface integrity of colistin-resistant bacterial strains, maintaining antibiotic potentiation with other drugs in pathogens expressing chromosomally mediated resistance to colistin monotherapy (Vidaillac et al., 2012). A previous study has shown that *mcr-1*-mediated LPS modification associated with colistin resistance can protect the cytoplasmic but not the outer membrane from damage caused by colistin. Despite OM damage of resistant bacteria, it was unable to kill or lyse them, leading to bacterial survival after treatment with colistin (Sabnis et al., 2021). Furthermore, permeabilization of the OM of colistin-resistant strains was sufficient to sensitize bacteria to other antibiotics as rifampicin, which normally cannot cross the OM (Humphrey et al., 2021). Whilst OM permeabilization is crucial for access of colistin to the cytoplasmic membrane, permeabilization of phospholipid bilayers of cytoplasmic membrane is required for the bactericidal and lytic activity of the antibiotic (Sabnis et al., 2021). The same can be applied to PMBN. Although all the strains tested in this study expressed high MICs to PMBN (64- ≥128 μg/ml) indicating that the molecule has no bactericidal effect when used alone, it was obvious that the molecule can perturb the integrity of the bacterial OM as seen by TEM. Morphological changes caused by exposure to PMBN were observed by other investigators using scanning electron microscopy (Dixon and Chopra, 1986). As observed in this study, PMBN induced numerous protrusions or blebs on the surface of *E. coli.* However, PMBN failed to cause leakage of the cytoplasmic components and the damage was confined to the OM, as reported before (Dixon and Chopra, 1986).

Furthermore, studies with 1-N-phenylnaphthylamine (NPN), a validated marker for outer membrane permeability of GNB verified that PMBN can increase outer membrane permeability in *E. coli* (Helander and Mattila-Sandholm, 2000). NPN is a hydrophobic fluorophore, which acts as a sensitive probe for OM barrier function. It can be excluded from the OM of intact cells, but if the OM is permeabilized, NPN enters the cells and emits fluorescence in the hydrophobic interior of the membrane, in a concentration-dependent manner (Akhoundsadegh et al., 2019). The same fluorescence pattern was noticed in this study as NPN fluorescence (% uptake) was more in the presence of high concentrations of PMBN and was reduced at low doses of the molecule. This dose-dependent effect can explain why PMBN produced potent bactericidal effects at high concentration (16–32 μg/ml) requiring lower doses of AZT to achieve bacterial killing, while it was less potent when applied at lower doses (2–8 μg/ml) requiring higher AZT concentrations to achieve the same effect as observed in the time-kill studies. Based on the results, it is evident that PMBN acted by increasing the permeability of the bacterial membrane facilitating the entry of AZT into the cell where it can exert its antimicrobial effects. Furthermore, AZT uptake was affected by the extent of OM damage induced by PMBN which is dose dependent.

It was noticed that strains with low MICs of AZT demonstrated better response to treatment with the combination compared to strains with high MICs. This is related to the genomic content of the strains. It is important to mention that more than half (62.96%) of the bacteria tested were resistant to AZT due to the presence of *mphA* with or without *ermB*. In this study, synergetic combinations were not effective in the presence of *ermB* gene. This gene encodes a methylase that confers macrolide-lincosamide-streptogramin B resistance. Methylation of the ribosomal target of the antibiotics by *ermB* enzyme causes resistance due to drug target alteration, resulting in the inability of AZT to interact with ribosome (Leclercq, 2002; Gomes et al., 2017). Thus, even if AZT is present in high concentration in the bacterial cytoplasm, it will not bind to the methylated target on the bacterial ribosome; thus, it could not inhibit protein synthesis. On the other hand, synergistic combinations were successful in the presence of *mphA* gene alone. This gene encodes the macrolide 2′-phosphotransferase I (MphA) which is an enzyme that causes inactivation of macrolides (Leclercq, 2002). It has been identified as a cause of resistance in *E. coli* (Xiang et al., 2020) and reported to be the most common gene associated with AZT resistance (Gomes et al., 2019), as was also observed in this study. A possible explanation on how the synergistic combinations were still effective in the presence of *mphA* gene is that when the intracellular concentration of AZT was high due to the OM permeabilization by PMBN facilitating drug entry, AZT was able to overpower the enzyme produced by the bacteria to inactive the drug. This effect may be augmented by the damage induced by PMBN, confirming the role of the synergistic combinations in causing bacterial death which is not achievable using a single agent.

It is noteworthy that *ermB* containing strains had higher AZT MICs (>128 μg/ml) than majority of *mphA* harboring strains (MIC: 32 to >128 μg/ml), which could affect the likelihood of success of synergy with PMBN. Unfortunately, the sole effect of *ermB* gene could not be explored as none of the strains tested in this study possess this gene alone, which is a common limitation encountered in previous studies (Tuan-Anh et al., 2020). The latter study reported that strains harboring both *ermB* with *mphA* genes had significantly higher MICs than strains without *ermB* gene, in agreement with our results. Moreover, Tuan-Anh et al. found that amongst the *E. coli* strains carrying *mphA* gene, strains with additional *ermB* gene demonstrated lower growth rate than strains devoid from this gene, which was attributed to reduced ribosomal synthesis in the presence of *ermB* gene due to methylation of 23S rRNA with potential interference with ribosome assembly. These observations support our conclusion that *ermB* gene has a substantial impact on bacterial susceptibility to AZT; therefore, it was associated with higher MICs and lack of response to the synergistic combinations. Furthermore, our results shed the light on the challenges facing the use of synergistic combinations on strains expressing multiple resistance mechanisms to the same antibiotic.

Indeed, the feasibility of using the synergistic combinations as therapeutic agents needs in-depth investigations. Combinations constituting of low dose of AZT may have promising clinical applications in treating different types of infections caused by *E. coli* when the last resort drugs are not effective. Clinically, *E. coli* is a common cause of various infections like UTIs (Foxman, 2010), bloodstream and gastrointestinal infections (Kaper et al., 2004). It is noteworthy that AZT is commonly used to treat diarrhea caused by some strains of *E. coli* (DuPont, 2007; Denham et al., 2018) with limited clinical applications in managing other types of infection. The combinations described in this study can expand the clinical spectrum of AZT to include other types of infections caused by *E coli*, but *in vivo* studies must be conducted to test the efficacy of these combinations. Clinical data are required to establish susceptibility breakpoints for AZT in *E. coli*, in order to guide the appropriate use of this antimicrobial agent with valuable biological activities.

## Conclusion

In this study, PMBN was able to re-sensitize *E. coli* to AZT, even in strains with high MICs when the drug used alone. The combinations were effective in the absence of genes that cause drug target alteration. Indeed, these findings highlight the importance of bacterial genotyping before applying therapeutic combinations, as some genes can cause irreversible resistance to certain antibiotics. We have demonstrated permeabilization of the outer membrane of *E. coli* by PMBN facilitating the entry of AZT molecule. To the best of our knowledge, the use of PMBN in synergy with AZT was not reported before, and as PMBN has good safety profile with less renal toxicity compared with colistin (Brown and Dawson, 2017), it can be tested in combination with other antibiotics, to explore the potential for using it at as an adjuvant to existing antibiotics. Further studies are warranted to evaluate if these combinations can act on other GNB. The same approach can be also used to enhance the entry of drugs with poor penetration into GNB membranes. Ultimately, this should be followed by testing the combinations *in vivo.* Indeed, synergistic drug combinations will be of added value to the exciting arsenal of antimicrobial agents in the war against MDR bacteria.

## Data availability statement

The original contributions presented in the study are included in the article/Supplementary material, further inquiries can be directed to the corresponding author.

## Author contributions

FA-M designed the experiments and obtained the funding. TC provided the clinical strains used in the study. AG, ST, and LD performed the experiments and preliminary analysis. FA-M analyzed the results and wrote the manuscript. All authors contributed to the article and approved the submitted version.

## Funding

This research was supported by grants from United Arab Emirates University (grant number G00003542 and G00003430).

## Conflict of interest

The authors declare that the research was conducted in the absence of any commercial or financial relationships that could be construed as a potential conflict of interest.

## Publisher’s note

All claims expressed in this article are solely those of the authors and do not necessarily represent those of their affiliated organizations, or those of the publisher, the editors and the reviewers. Any product that may be evaluated in this article, or claim that may be made by its manufacturer, is not guaranteed or endorsed by the publisher.

## Supplementary material

The Supplementary material for this article can be found online at: https://www.frontiersin.org/articles/10.3389/fmicb.2022.998671/full#supplementary-material
