# Supplementary material for: Discerning the role of polymyxin B nonapeptide in restoring the antibacterial activity of azithromycin against antibiotic-resistant Escherichia coli
Source: Front Microbiol. 2022 Sep 21;13:998671. doi: 10.3389/fmicb.2022.998671 (PMC9532765; doi:10.3389/fmicb.2022.998671)
Supplement: Supplementary file 2 [file Data_Sheet_1.docx]

**Supplementary Figure 1.** Time kill graph for strain BAA-2469 tested with different synergistic combinations of PMBN (32- 2 µg/ml) and azithromycin (AZT) (32- 2 µg/ml). A single agent (AZT or PMBN) at a concentration of 32 µg/ml were used as controls in addition to an untreated growth control (GC). AZT ≥ 4 µg/ml killed the bacteria in all the synergistic combinations with PMBN. Mean of duplicates from 2 independent experiments ± SD are shown.

**Supplementary Figure 2.** Time kill graph for strain EC24 tested with different synergistic combinations of PMBN (32- 2 µg/ml) and azithromycin (AZT) (4- 0.5 µg/ml). A single agent (AZT or PMBN) at a concentration of 4 and 32 µg/ml, respectively were used as controls in addition to an untreated growth control (GC). AZT ≥ 0.5 µg/ml killed the bacteria in all the synergistic combinations with PMBN. Mean of duplicates from 2 independent experiments ± SD are shown.

**Supplementary Figure 3.** Time kill graph for strain EC477 tested with different synergistic combinations of PMBN (32- 2 µg/ml) and azithromycin (AZT) (32- 2 µg/ml). A single agent (AZT or PMBN) at a concentration of 128 and 32 µg/ml, respectively were used as controls in addition to an untreated growth control (GC). AZT ≥ 4 µg/ml killed the bacteria in all the synergistic combinations with PMBN. Mean of duplicates from 2 independent experiments ± SD are shown.

**Supplementary Figure 4.** Time kill graph for strain EC26 tested with different synergistic combinations (A-E) of PMBN (32- 2 µg/ml) and azithromycin (AZT) (64- 4 µg/ml). A single agent (AZT or PMBN) at a concentration of 128 and 32 µg/ml, respectively were used as controls in addition to an untreated growth control (GC). AZT ≥ 4 µg/ml killed the bacteria in all the synergistic combinations with PMBN. Figure F shows the effect of AZT when used alone on the bacterial strain, whereby AZT was not able to kill the bacteria at concentrations below the MIC (128 µg/ml). Mean of duplicates from 2 independent experiments ± SD are shown.
